# Anti-*Candida* Biofilm Activity of Pterostilbene or Crude Extract from Non-Fermented Grape Pomace Entrapped in Biopolymeric Nanoparticles

**DOI:** 10.3390/molecules24112070

**Published:** 2019-05-30

**Authors:** Giovanna Simonetti, Cleofe Palocci, Alessio Valletta, Olga Kolesova, Laura Chronopoulou, Livia Donati, Antonio Di Nitto, Elisa Brasili, Pierpaolo Tomai, Alessandra Gentili, Gabriella Pasqua

**Affiliations:** 1 Department of Public Health and Infectious Diseases, “Sapienza” University of Rome, P. le Aldo Moro 5, 00185 Rome, Italy; giovanna.simonetti@uniroma1.it (G.S.); olga.kolesova@uniroma1.it (O.K.); 2 Department of Chemistry, “Sapienza” University of Rome, 00185 Rome, Italy; cleofe.palocci@uniroma1.it (C.P.); laura.chronopoulou@uniroma1.it (L.C.); antonio.dinitto@uniroma1.it (A.D.N.); pierpaolo.tomai@uniroma1.it (P.T.); alessandra.gentili@uniroma1.it (A.G.); 3 Department of Environmental Biology, “Sapienza” University of Rome, 00185 Rome, Italy; alessio.valletta@uniroma1.it (A.V.); donatilivia@gmail.com (L.D.); elisa.brasili@uniroma1.it (E.B.)

**Keywords:** *Candida albicans* biofilm, biopolymeric nanoparticles, *Vitis vinifera*, crude grape pomace extract, pterostilbene, antifungal activity

## Abstract

Polymeric nanoparticle-based carriers are promising agents to deliver drugs to cells. *Vitis vinifera* phenolic compounds are known for their antifungal activity against *Candida albicans*. The aim of the present study was to investigate the antifungal activity of pterostilbene or crude extracts from non-fermented grape pomace, entrapped in poly(lactic-co-glycolic) acid nanoparticles (NPs), with diameters of 50 and 150 nm, on *Candida* biofilm. The fluorescent probe coumarin 6 was used to study the uptake of poly(lactic-co-glycolic)acid (PLGA) NPs in planktonic cells and biofilm. The green fluorescent signal of coumarin 6 was observed in *Candida* biofilm after 24 and 48 hours. Both pterostilbene and crude pomace extract entrapped in NPs exerted a significantly higher anti-biofilm activity compared to their free forms. The entrapment efficiency of both pterostilbene and crude pomace extract in PLGA NPs was ~90%. At 16 µg/mL, pterostilbene loaded in PLGA NPs reduced biofilm formation of 63% and reduced mature biofilm of 50%. Moreover, at 50 µg/mL, the pomace extract loaded in NPs reduced mature biofilm of 37%. These results strongly suggest that PLGA NPs are promising nanodevices for the delivery of antifungal drugs as the crude grape pomace extract, a by-product of white wine making.

## 1. Introduction

*Candida albicans* is an opportunistic pathogen, part of the normal microbiota of the human oral cavity, gastrointestinal tract and female genital tract. *C. albicans* infections range from superficial mucosal and dermal infections to disseminated bloodstream infections with mortality rates above 40% [1,2]. The medical impact of *C. albicans* is typically due to its ability to form biofilms which are closely packed communities of cells adhering to surfaces and embedded in a protective polymeric extracellular matrix. Treatment of *C. albicans* biofilms with drugs currently on the market represents a significant clinical problem due to the intrinsic tolerance of fungal biofilms to many anti-fungal agents [3]. To fight *Candida* biofilms, high doses and frequent administrations of antifungal drugs are required, causing adverse side effects or in some cases toxicity [4].

Since few molecules are reported in the literature to inhibit or prevent the formation of *C. albicans* biofilms, the development of new anti-biofilm agents represents an important field of investigation. Compounds of natural origin, i.e., plant-derived molecules, could represent a new strategy for the prevention of fungal adhesion and biofilm formation [5,6]. Some authors have demonstrated that certain essential oils inhibit biofilms formed by *Candida* spp. [7,8,9,10,11,12,13,14,15], however, their toxicity to biological membranes is also known [16].

Extracts obtained from grape pomace are particularly interesting and represent a rich source of various high-value products, characterized by the presence of several bioactive phenolic compounds, including *trans*-resveratrol and other stilbenes, quercetin, proanthocyanidins, anthocyanins, hydroxybenzoic and hydroxycinnamic acids, and catechins [17].

Among stilbenes, *Vitis* inducible phytoalexins, pterostilbene (PTB) is one of the most active antifungal compounds, even more than resveratrol and viniferins [10]. Li et al. [18] reported that PTB has a strong in vitro and in vivo activity against *C. albicans* biofilm. They also demonstrated the antifungal activity of PTB against plant fungal pathogens, such as *Phomopsis viticola*, *P. obscurans*, and *Botrytis cinerea.*

To increase the drug efficacy and decrease the amount of antifungals administered, recent studies report the use of antifungals loaded into nanovectors [19,20]. The use of nanobiotechnology could be a new and promising strategy to fight microorganisms. Drug incorporation into nanoparticles (NPs) could greatly reduce drug toxicity. Moreover, rationally designed drug delivery systems can improve drug performance [21]. Poly(lactic-co-glycolic) acid (PLGA) NPs represent promising drug carriers. Recently, we demonstrated that PLGA NPs are able to penetrate into some fungi such as *Aspergillus carbonarius*, *A. niger* and *B. cinerea* [22,23]. Moreover, a microfluidic system to deliver ribavirin using PLGA NPs has been studied by us [24]. Yang et al. (2019) reported the synergistic antifungal effect of amphotericin b-loaded poly (lactic-co-glycolic acid) nanoparticles of 220–290 nm and ultrasound [25].

In our study, for the first time, PLGA NPs were loaded with PTB or crude extract from non-fermented pomace of *V. vinifera* cv. Italia, to investigate the anti-biofilm activity on four *C. albicans* strains. The choice to use PTB starting from the concentration of 16 µg/ml is based on a recent literature finding [18].

## 2. Results 

### 2.1. Physicochemical Characterization of PLGA NPs Loaded With PTB and Pomace Extracts

The dimensions of PTB or extract-loaded PLGA NPs are reported in Table 1. PLGA NPs, entrapping PTB or crude pomace extracts, according to the experimental conditions used, presented a size of 50 and 150 nm, with polydispersity indexes below 0.5. Investigations by scanning electron microscopy (SEM) showed that drug-loaded PLGA NPs had a spherical morphology. The drug loading of PTB within PLGA NPs was of 145 µg/mg of PLGA, corresponding to an encapsulation efficiency of 90%. A similar entrapment efficiency was obtained for pomace with a value of 87.2%.

### 2.2. Chemical Characterization of Pomace Extracts

The total polyphenols and procianydins observed in the pomace extracts were 35.85 mg/g DW of gallic acid equivalent and 90.67 mg/g DW, respectively. The metabolites identified in pomace extract by HPLC-ESI-MS are reported in Table 2. The TIC chromatograms of the pomace extract before and after NPs entrapment were reported in Figure S2A,B. The pomace extract was characterized by the presence of caffeic acid, catechin/epicatechin, gallic acid, procyanidins, quinic acid and a series of unknown compounds.

### 2.3. PLGA NPs Localization in C. albicans Planktonic Cells and Biofilm

**Figure 1:**
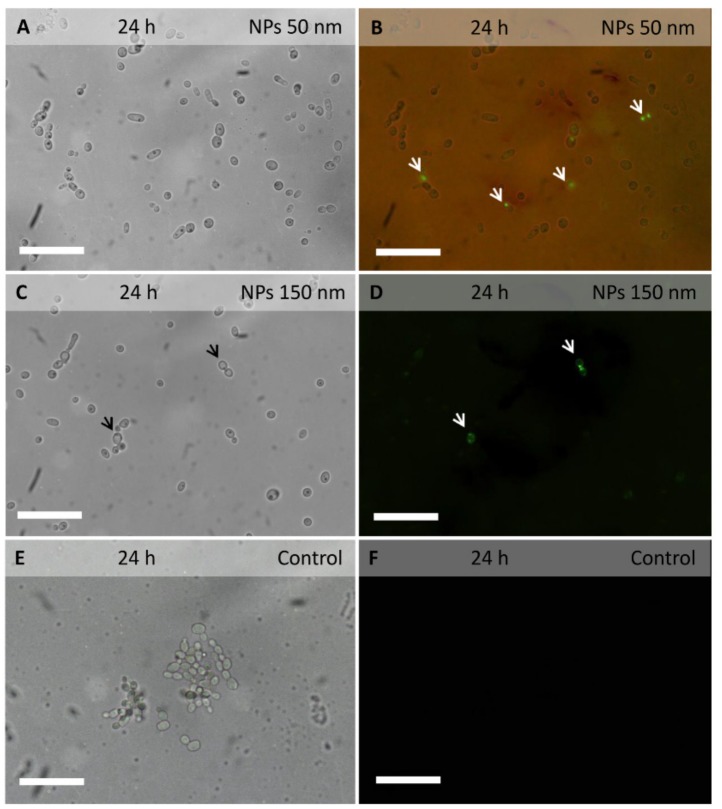
*Candida albicans* planktonic cells observed under bright field (**A**,**C**,**E**) or under fluorescence (**B**,**D**,**F**) after 24 h of growth incubated for 3 h with coumarin 6-loaded PLGA NPs. The fluorescent signal was observed in several cells incubated with NPs of 50 nm in diameter (arrows in **B**) and in a few cells incubated NPs of 150 nm in diameter (arrows in **D**). In control cells no fluorescent signal was observed (**F**). Scale bars represent 40 µm.

**Figure 2:**
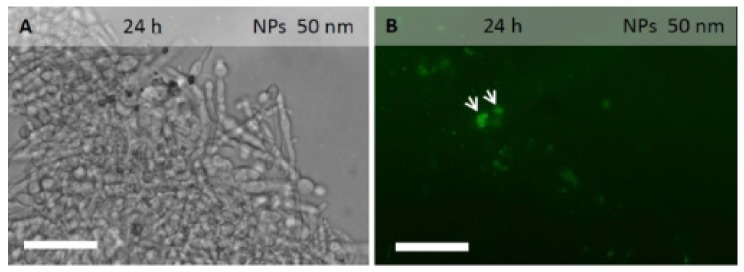

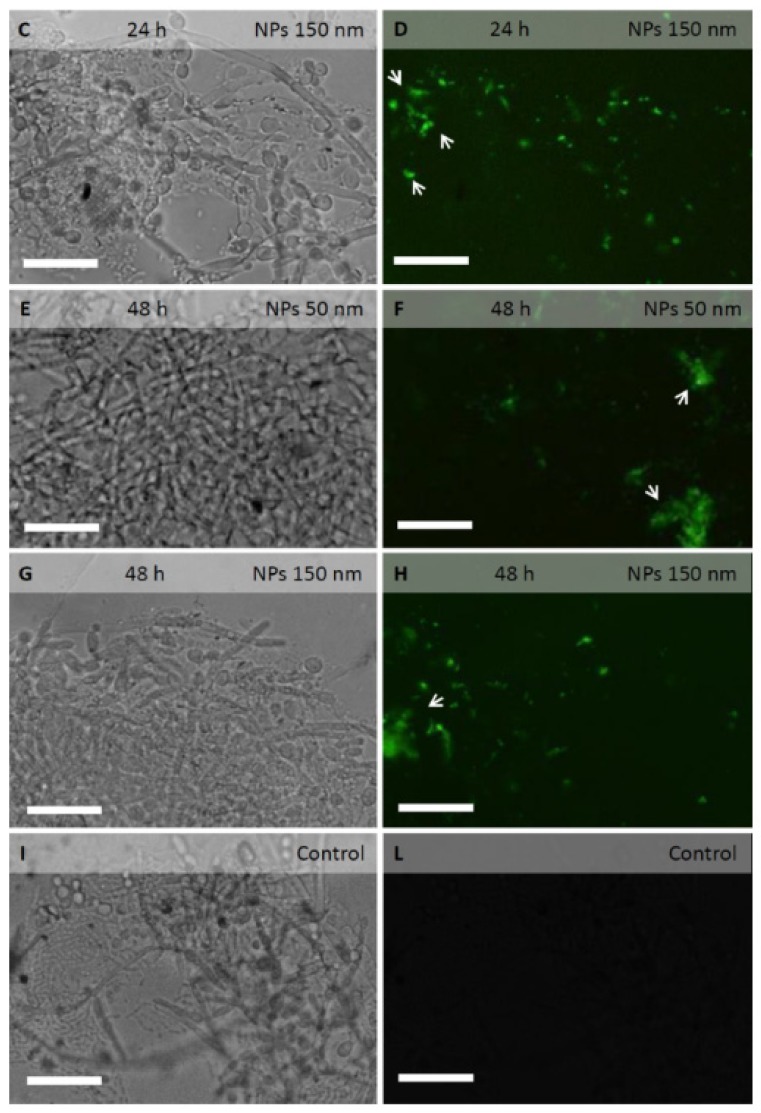
*C. albicans* biofilm grown on contact lenses for 24 h (**A**–**D**) or 48 h (**E**–**H**), incubated for 3 h with coumarin 6-loaded PLGA NPs of 50 nm (**A**–**B**,**E**–**F**) or 150 nm (**C**–**D**,**G**–**H**) in diameter, washed 3 times and observed in bright field (**A**,**C**,**E**,**G**) or under fluorescence (**B**,**D**,**F**,**H**). Independently from NP dimensions, the fluorescent signal was frequently observed into the biofilm. Scale bars represent 20 µm.

Fluorescent coumarin 6-loaded PLGA NPs were added to *C. albicans* cell cultures to investigate their localization in planktonic cells, as well as in biofilm extracellular matrix and cells (Figure 1 and Figure 2).

The green fluorescent signal of coumarin 6 was observed within several planktonic cells incubated for 3 h with PLGA NPs of 50 nm in diameter (Figure 1A,B). Even with PLGA NPs of 150 nm in diameter (Figure 1C,D), the fluorescent signal was observed within planktonic cells, though more rarely.

In biofilm of 24 hours, incubated for 3 h with PLGA NPs of 50 or 150 nm in diameter, the fluorescent signal was observed within the extracellular matrix and around the cells. The signal was still visible even after numerous rinses, indicating a strong adhesion of PLGA NPs to the biofilm extracellular matrix and/or cell walls. The signal was also observed within several hyphal cells, suggesting the ability of PLGA NPs to cross the cell wall and membrane (Figure 2B,D,F,H).

At 48 hours post-inoculum, the biofilm was more structured. After incubation with PLGA NPs of 50 or 150 nm in diameter, the fluorescent signal was visible in the extracellular matrix, around the hyphal cells and within several hyphal cells (Figure 2F,H).

### 2.4. Antifungal Activity of PTB and Pomace Extract-Loaded PLGA NPs on C. albicans Planktonic Cells

The activity of PTB and pomace extract, free and loaded within PLGA NPs, was evaluated against *C. albicans* planktonic cells. No antifungal activity for PTB, neither free or loaded PLGA NPs, was found against *C. albicans* planktonic cells, at concentrations ≤16 µg/mL (Table 3).

Differently from PTB, pomace crude extract showed antifungal activity on planktonic cells with GM MIC_50_ values of 17.76 µg/mL. Pomace crude extract-loaded NPs showed lower activity than the free crude extract, with GM MIC_50_ value of 50 µg/mL. No 100% of inhibition was found at concentration ≤50 µg/mL.

### 2.5. Antifungal Activity of PTB and Pomace Extract-Loaded PLGA NPs on C. albicans Biofilm

**Figure 3:**
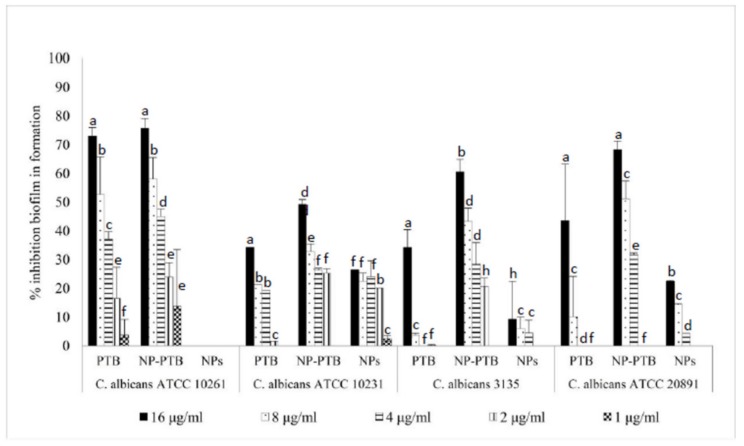
Activity of pterostilbene, free and loaded in PLGA NPs, against *Candida albicans* in formation biofilm. The values are expressed as mean ± standard deviation from three independent experiments in duplicate. One-way ANOVA followed by Dunnett’s test was used for statistical significance. Different letters indicate statistically significant differences between samples (*P* < 0.05).

**Figure 4:**
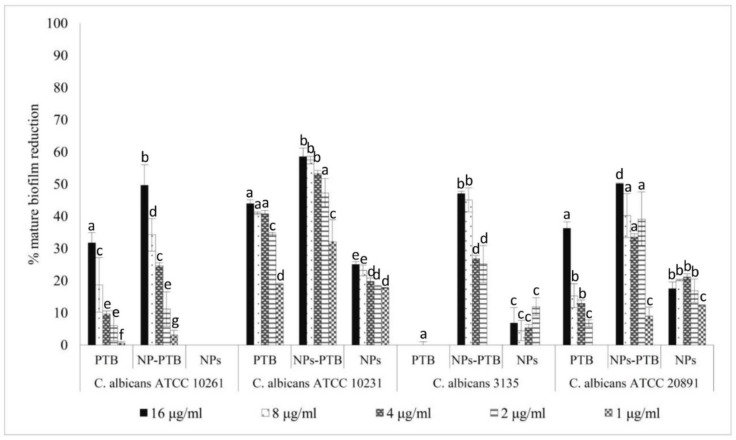
Activity of pterostilbene, free and loaded in PLGA NPs, against *Candida albicans* mature biofilm. The values are expressed as mean ± standard deviation from three independent experiments in duplicate. One-way ANOVA followed by Dunnett’s test was used for statistical significance. Different letters indicate statistically significant differences between samples (*P* < 0.01).

The activity of PTB, both free and loaded within PLGA NPs, was evaluated against *C. albicans* biofilm formation and against mature biofilm. The results reported in Figure 3 and Figure 4 show that PTB-loaded PLGA NPs inhibited biofilm formation and reduced mature biofilm, with a biofilm inhibition concentration of 50% (BMIC_50_) of 16 µg/mL. The results showed also a higher activity of PTB-loaded PLGA NPs than free PTB against *C. albicans* biofilm formation (*p* < 0.05) in two *C. albicans* strains (Figure 3). The mature biofilm reduction with PTB-loaded PLGA NPs was significantly greater than with free PTB in all the strains (Figure 4).

**Figure 5:**
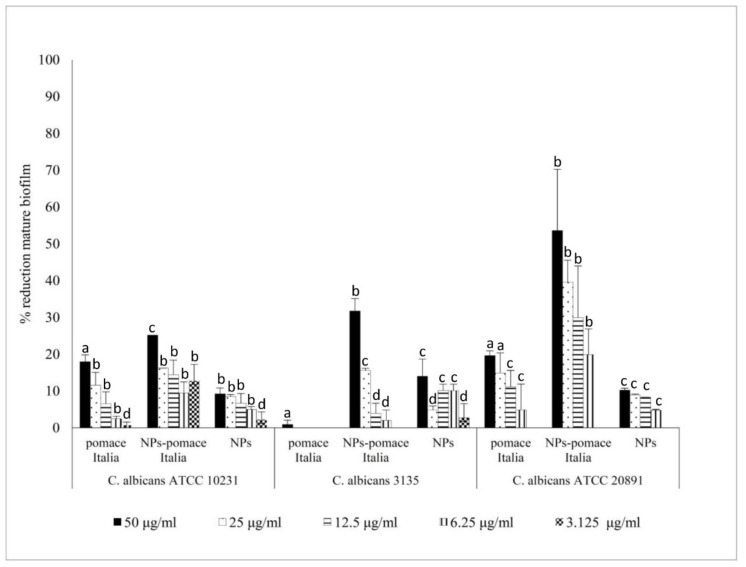
Activity of pomace extract, free and loaded in PLGA NPs, against *Candida albicans* mature biofilm. The values are expressed as mean ± standard deviation from three independent experiments in duplicate. One-way ANOVA followed by Dunnett’s test was used for statistical significance. Different letters indicate statistically significant differences between samples (*P* < 0.01).

Moreover, a better activity of pomace extract-loaded PLGA NPs against mature biofilm than free pomace extract has been demonstrated (Figure 5). Pomace extract-loaded PLGA NPs, at 50 µg/mL, destroyed the mature biofilms of 37%. The best results were obtained on *C. albicans* ATCC 20891. The results of pomace extract loaded PLGA NPs showed a BMIC_50_ of 50 µg/mL against *C. albicans* ATCC 20891 mature biofilm.

## 3. Discussion

Fungal pathogens form biofilms that are highly recalcitrant to antimicrobial therapy. *C. albicans* biofilm forms when single cells attach to a surface. Addition of a *C. albicans* culture to a solid surface initiates a 60–90 minutes adherence phase. The next stage in biofilm development consists of cell proliferation and early-stage filamentation of the adhered cells. This is followed by biofilm maturation resulting in a complex three-dimensional structure that is held together by hyphae and an exopolymer matrix. Mature *C. albicans* biofilm forms after 24 h of incubation and it is a network of yeasts, hyphae, pseudohyphae, and extracellular material [26,27]. *C. albicans* biofilm constitutes a threat to successful antifungal treatment. Several authors reported that biofilm resistance to antimicrobials is a multifactorial mechanism involving planktonic antifungal resistance and biofilm-specific mechanisms. One biofilm-specific mechanism involves the complex extracellular matrix. Components of the matrix, specifically β-glucan, mannan, and extracellular DNA, have been found to promote resistance against multiple antifungal drug classes [28,29]. The strategies to fight *Candida* biofilm, that overcome the resistance given by the matrix, require the use of novel approaches.

In recent years, micro and nanostructured polymeric materials, due to their peculiar chemico-physical characteristics, gained considerable interest in many research areas especially as promising tools for a variety of biotechnological applications, such as drug delivery vectors to target fungi or bacterial cells responsible for mammalians infections. Ag NPs have proven to be very effective, as they have shown antimicrobial efficacy against bacteria, viruses and other eukaryotic microorganisms [30]. However, toxicity from Ag NPs has been observed. Moreover, their small size and variable properties most probably make them hazardous to the environment [31].

Biopolymers are usually preferred due to their biocompatibility, biodegradability and non-toxicity. PLGA NPs are currently considered among the most promising drug carriers [32]. The main advantages in using nanopolymeric vectors to fight biofilm formation and developing are: i) ability to penetrate within the extracellular matrix and cells; ii) controlled release of the active molecules that prolong the exposure of the pathogen to the antifungal and lower toxicity; iii) protection of the load from chemico-physical degradation and biodegradation (especially for polyphenolic compounds due to their intrinsic instability). NPs have been recently applied to treat cystic fibrosis-associated *Pseudomonas aeruginosa* biofilm [19]. In this work, PLGA was selected as polymeric matrix based on its large use in several biomedical applications for drug delivery such as vaccination, cancer, inflammation and other diseases, as well as for its low cost and wide availability [33]. By using a microfluidic method, PLGA NPs of 50 and 150 nm entrapping a fluorescent probe (coumarin 6) were synthesized to investigate their ability to cross the biofilm matrix or the fungal yeast cell wall. To the best of our knowledge, no data have been published on the uptake of PLGA NPs in *Candida* biofilm.

Epifluorescence analysis suggested that PLGA NPs are able to penetrate *Candida* yeast and hyphal cells. Experiments on *Candida* biofilm showed that PLGA NPs penetrate the extracellular matrix and cells. In a previous study, the cellular uptake of PLGA NPs we observed in hyphal cells of *A. carbonarius*, *A. niger* and *B. cinerea* [22].

Concerning molecules isolated from plants, few have shown anti-*Candida* biofilm activity. In 2008, thirty plant essential oils were tested for their activity against *C. albicans* biofilms and four of them, such as eucalyptus, peppermint, ginger grass and clove showed 80.87%, 74.16%, 40.46%, and 28.57% biofilm reduction, respectively [7]. Polyphenols extracted from green tea also proved to be active against *C. albicans* biofilm. Epigallocatechin-3-gallate, the most abundant polyphenol in green tea extract, reduced *C. albicans* biofilm formation [8]. Among the molecules isolated from *V. vinifera*, PTB has been shown to have activity against *Candida* biofilm [10].

In order to demonstrate if PLGA NPs loaded with phytoalexins were able to increase their antibiofilm activity, we chose PTB as a model molecule and we entrapped it within nanosized particles. The synthetized NPs loaded with PTB showed an entrapment efficiency equal to 90% and a significant activity both against *Candida* biofilm formation and *Candida* mature biofilm. At the concentration of 8 µg/mL, PTB alone reduced mature biofilm of 5% and inhibited biofilm formation of 14%. Conversely, PTB-loaded PLGA NPs reduced mature biofilm of 43% and inhibited biofilm formation of 45%.

Yano et al. [34] reported that a phenolic fraction from the pomace of *V. coignetiae* inhibited biofilm formation by *Streptococcus mutans*. To the best of our knowledge, no data have been published on the anti-*Candida* biofilm activity of grape pomace extracts. In this study, it was observed that grape pomace extracts loaded within PLGA NPs with high entrapment efficiency (87.2%), reduced mature biofilms of *C. albicans* ATCC 20891 of 50% at 50 µg/mL. Non-fermented pomace extract from *V. vinifera* is known to contain a wide range of polyphenols such as anthocyanins, hydroxybenzoic acids, hydroxycinnamic acids, catechins, flavonols, stilbenes, and proanthocyanidins [35]. Previously, it has been demonstrated that the extracts from *V. vinifera* L. seeds obtained from mature grapes, rich in polymeric flavan-3-ols, exhibit good antifungal activity against *Candida* species, suggesting their use in muco-cutaneous fungal infections [11,12,13,14,15]. A correlation between proanthocyanidins and antifungal activity against *Candida* planktonic cells has also been demonstrated (11). The crude grape pomace extract used in the present study contained not only proanthocyanidins, but also other secondary metabolites and unidentified compounds therefore, a synergic effect of all metabolites on antifungal activity cannot be excluded.

## 4. Conclusions

Anti-*Candida* biofilm activity of PTB and crude grape pomace extract loaded PLGA NPs was higher than free formulations. PTB and grape pomace extract showed high bioavailability and no toxicity. In conclusion, entrapment a crude grape pomace extract, a by-product of white wine making, into PLGA NPs, could be considered an effective anti-*Candida* biofilm strategy and could be attractive for large scale use.

## 5. Materials and Methods

### 5.1. Plant Material and Pomace Extract Preparation

Plants of *V. vinifera* L. cv. Italia were grown in the experimental farm of Crea-Utv, Turi (BA, Italy) and the grapes harvested at technological maturation and put in small boxes (30 × 40 cm), 5 kg of weight each, as previously described [11]. The bunches were destemmed and pressed, and the obtained non-fermented pomace was weighed, immediately frozen and stored at −20 °C. The non-fermented pomace was put in liquid nitrogen in a porcelain mortar and grinded to obtain a fine powder. It was extracted three times (24 h for each extraction) with EtOH/H_2_O (7:3 *v*/*v*) and acidified with formic acid at pH 3; the ratio matrix/solvent was 1 g/10 mL. The extract was dried by using a rotavapor at 30 °C, then dissolved in EtOH 70% with a solvent/dry extract ratio of 1/1 (mg D.W./mL) and filtered through a 0.45 μm Minisart filter (Sartorious, Goettingen, Germany).

### 5.2. NP Preparation and Characterization

PLGA NPs loaded with PTB, pomace extracts and coumarin 6 were prepared by using a microfluidic reactor with a previously reported flow-focusing configuration [36] and outlined in Figure S1 (Supplementary Materials). Fluorescent coumarin 6-loaded PLGA NPs were added to *C. albicans* cell cultures to investigate their localization in planktonic cells, as well as in biofilm extracellular matrix and cells. Coumarin 6 was chosen because it is a stable fluorescent probe and it is commonly employed in uptake studies of nanomaterials [23].

To this aim, the polymer (2 mg/mL) and the selected payload were dissolved in an organic phase. To dissolve PTB (0.5 mg/mL) and pomace crude extract (1 mg/mL) acetone and DMSO were used, respectively. Each organic solution was injected in a continuous flow microfluidic reactor system as described by Palocci et al. [23]. All experiments were conducted in triplicate and no precipitate was observed in the micro-channels during flow-focusing experiments. The formed NPs were recovered, the organic phase was eliminated under reduced pressure, then the aqueous suspensions were sonicated and stored at −20°C until their use. To synthetize PLGA NPs with diameters of 50 and 150 nm the aqueous flow rate was kept constant at 2000 µL/min while the organic phase flow rate was varied between 100 and 400 μL/min. Poly-(d,l-Lactic-co-glycolic) acid (PLGA) 50:50, MW 50 KDa, coumarin 6, DMSO, acetone (99%), ultrapure water were purchased from Sigma-Aldrich (Milan, Italy).

Dynamic light scattering (DLS) measurements were carried out using a NanoZetasizer (Malvern Instruments, Malvern, UK) to evaluate the mean diameter of PLGA NPs and their polydispersity index. The experimental conditions used are the following: a helium neon laser operating at 633 nm, a fixed scattering angle of 173 degrees and constant temperature (25 °C). The measured autocorrelation functions of the scattered light intensity were analyzed using the CONTIN algorithm in order to obtain the decay time distributions [37]. Decay times were used to determine the distributions of the diffusion coefficients of the particles (D), converted in turn in the distributions of the apparent hydrodynamic radii, R_H_, using the Stokes-Einstein relationship: R_H_ = k_B_T/6πηD (k_B_T = thermal energy; η = solvent viscosity). Particle morphology was observed by scanning electron microscopy (SEM) in both the secondary and the backscattered electron modes with an electron acceleration voltage of 20 keV, using a LEO 1450VP SEM microscope (ZEISS, Oberkochen, Germany).

### 5.3. Chemical Characterization of Non-Fermented Grape Pomace

The non-fermented pomace extract was analyzed spectrophotometrically (Beckman DU Series 600 spectrophotometer, Beckman Coulter, Brea, CA, USA) for the quantification of total polyphenols through Folin-Ciocalteu method, as previously described by Slinkard and Singleton [38].

The quantification of oligomeric and polymeric procyanidins was determined by HPLC (Waters 1525, Waters detector 2487 and a Waters C_18_ 4.6 mm × 250 mm Symmetry^®^ column, 5 µm porosity, Waters, Milford, MA, USA), as previously described by Simonetti et al. [11]. Two defensive phytoalexin molecules, procyanidin B2 and PTB standards of high purity grade, were purchased from Extrasynthese (Genay, France) and Sigma-Aldrich, respectively.

Aliquots of total crude and non-entrapped pomace extract were analyzed using a Perkin Elmer series 200 binary pump equipped with an autosampler (Perkin Elmer, Norwalk, CT, USA). The separation was carried out on a Waters XTerra C_18_ (3.5 μm) column (2.1 × 150 mm) protected by a guard column. The mobile phases were water (phase A) and CH_3_CN (phase B) both 0.1% in formic acid at a constant flow–rate of 0.2 mL/min. The linear gradient elution profile was as follows (t in min): t0, B = 0%; t5, B = 0%; t45, B = 25; t60, B = 100%; t70, B = 100%. The injection volume was 2 μL. After each injection, the autosampler needle was washed with CH_3_CN. Chromatograms were acquired with a triple quadrupole mass spectrometer PE-Sciex API-3000^®^ (Perkin Elmer Sciex Toronto, ON, Canada), equipped with an electrospray source (ESI) operated in negative ionization. The capillary voltage was −4500 V. High purity nitrogen was used as curtain and collision gas, while air was employed as nebulizer and drying gas. The latter was heated by setting the source heather temperature at 350 °C. The full width at half maximum (FWHM) was set at *m*/*z* 0.7 ± 0.1 in each mass-resolving quadrupole to operate with a unit resolution. The mass spectrometer was operated in Full Scan mode in a mass spectral range as 150–800 *m*/*z*. HPLC–MS data were acquired and elaborated by Analyst^®^ 1.5 Software (AB Sciex).

### 5.4. Determination of Entrapment Efficiency of PTB and Pomace Extract

The quantitative analysis of PTB entrapped within PLGA NPs was carried out by spectroscopic measurements. NPs aqueous suspensions were ultra-centrifuged at low temperature to recover NPs. NPs were dissolved in acetone and analyzed by measuring the UV absorbance at 283 nm and comparing the results with a calibration curve. To verify the entrapment efficiency of the pomace extract in PLGA NPs, an analysis of the pomace extract before and after its entrapment in PLGA NPs was carried out by HPLC-ESI-MS spectrometry. The entrapment efficiency in PLGA NPs was calculated as percent ratio of the area of grape pomace extract entrapped to the residual amounts of non-entrapped extract.

### 5.5. Antifungal Susceptibility Testing

To evaluate the minimal inhibitory concentration (MIC), *C. albicans* ATCC 10231, ATCC 20891, ATCC 10261 and 3153A strains from the American Type Culture Collection (Rockville, MD, USA) were tested. The broth microdilution method to evaluate the susceptibility in vitro on *C. albicans* strains was performed according to a standardized method for yeasts [39,40] with some modifications for natural products. *C. albicans* strains were grown on Sabouraud dextrose agar (Sigma Aldrich, St. Louis, MO, U.S.A.) at 35 °C for 24 h. The final concentration of the inoculum was 1 × 10^3^–5 × 10^3^ cells/mL. The in vitro antifungal susceptibility was evaluated using pure PTB as a reference drug, and crude extracts of non-fermented pomace, free or loaded in PLGA NPs. PTB and pomace extracts were dissolved in DMSO (Sigma Aldrich) at concentrations at least 100 times higher than the highest desired test concentration. DMSO at 1% concentration without any dissolved substance was used as a control [39,40]. The final concentration of PTB ranged from 16 to 0.125 µg/mL, the final concentration of extracts ranged from 50 to 0.21 µg/mL. The MIC_50_, MIC_90_ and MIC_100_ were evaluated, which are the lowest concentrations of extracts or PTB that caused growth inhibitions ≥50%, ≥90% and 100%, respectively. The antifungal activities are the result of three independent experiments performed in duplicate.

### 5.6. In Vitro Biofilm Formation Assay

The anti-biofilm activity was evaluated as described by Pierce et al. [41]. The assay was performed in a 96-well tissue culture plate by seeding with 1.0 × 10^6^ cells/mL *C. albicans* cells in RPMI 1640 medium and incubating them statically at 37 °C. To detect the effect of PTB and pomace extract on the formation of biofilms, different concentrations of PTB and pomace extract, used in free form or loaded in PLGA NPs, were added to fresh RPMI 1640 after 90 min of adhesion. The plates were further incubated at 37 °C for 48 h. A mature biofilm has been visualized by eye as a cloudy surface structure on top of the solid surface, and under a microscope, as an organized collection of different cell types as described by Gulati and Nobile [42]. To detect the effect of PTB and pomace extract, free and loaded in PLGA NPs, on mature biofilms, *C. albicans* biofilms were formed at 37 °C for 24 h as described above. The biofilm supernatant was then discarded and fresh RPMI 1640 medium containing different concentrations of PTB and extracts, free or loaded in PLGA NPs, was added. The plates were incubated at 37 °C for an additional 24 h as previously described [41]. A semiquantitative measure of the formed biofilms was calculated with a 2,3-bis-(2-methoxy- 4-nitro5-sulfophenyl)-2*H*-tetrazolium-5-carboxanilide (XTT) reduction assay [41]. The Biofilm Minimal Inhibitory Concentration (BMIC) end point for biofilm was based on the lowest drug concentration producing a decrease of 50% metabolic activity relative to the untreated growth control (XTT reduction assay). At least two experiments were performed on two separate dates for each compound tested in triplicate.

### 5.7. Epifluorescence Microscopy Analysis

Epifluorescence microscopy analysis was performed to investigate if coumarin 6-loaded PLGA NPs adhered or entered in *C. albicans* planktonic cells and if they penetrated *C. albicans* biofilm matrix or cells. The localization of coumarin 6-loaded PLGA NPs was visualized with an epifluorescence microscope apparatus described elsewhere [13] fitted with a blue filter (*λ*_excitation_386 nm; *λ*_emission_ 490 nm). *C. albicans* cells were grown on Sabouraud dextrose agar (Sigma Aldrich) at 35 °C for 24 h. Then the cells were suspended into a saline solution with or without PLGA NPs and incubated for 3 h. The biofilm was grown on silicone–hydrogel contact lenses (Day Acuvue Moist, Johnson & Johnson, New Brunswick, NJ, USA). After 24 and 48 h of incubation at 37 °C, PLGA NPs were added and incubated for 3 h. Before the observation, the contact lenses were subjected to three washes in physiological solution to remove the non-adherent PLGA NPs from the biofilm.

### 5.8. Statistical analysis

Data were expressed as mean ± S.D. Experimental data were analyzed using GraphPad Prism 5.3 software (GraphPad Software, San Diego, CA, USA). The one-way analysis of variance (ANOVA) was used, followed by Dunnett’s test. The results were considered statistically significant at *P* < 0.05.

## Figures and Tables

**Table 1:** Size and PI of PLGA NPs, empty or loaded with PTB, crude plant extracts or coumarin 6.

PLGA NPs	NPs Size (nm) ± SD	PI ± SD
Empty PLGA NPs	50 ± 12	0.3 ± 0.03
Coumarin 6-loaded PLGA NPs	50 ± 14	0.4 ± 0.04
PTB-loaded PLGA NPs	50 ± 10	0.2 ± 0.02
Pomace crude extract-loaded PLGA NPs	50 ± 13	0.3 ± 0.03
Coumarin 6-loaded PLGA NPs	150 ± 14	0.2 ± 0.02

PLGA NPs = poly(lactic-co-glycolic) acid nanoparticles; PTB = pterostilbene; PI = polydispersity index; SD = standard deviation.

**Table 2:** Chemical characterization of methanolic extract from grape pomace.

*m*/*z*	Retention Time (min)	Metabolite	Fragments
178.9	2.1–3.3	Caffeic acid	58.7/70.6/89/85.1
289.1	30.5–30.9	Catechin/Epicatechin	109/124.8/203.1/151.1/245.2/123/161.1205.1/137/221.2/289/179/108.9/124.8/203.1/122.8/150.9/204.7/245.3/289
168.8	11–12.3 22.43	Gallic acid	125/168.6/79.1/80/96.8/107.1/125/169.1/78.8/ 97.2/106.9
577.1	28.6–29.8/32–33.2/33.4–33.8/34.7–38.4/42.8–43	Procyanidin	407.3/289.1/425.2/125/450.8/577
191	2.5–5.6	Quinic acid	110.8/72.7/85.1/58.8/98.9/117/111/86.8/85
195	1.3–2.5 5.6	Unknown	74.7/98.8/86.9/85.1/58.7/70.7/89/128.9/194.8
224.9	2.1–2.8/15.7	Unknown	89.1/58.7/70.6/112.8/100.9/85.1/118.8
161.1	2.2–2.5/19.5–20.1	Unknown	70.6/85/72.6/58.8/94.9/100.8/56.7/83.1/113.1
253.1	2.2–4.2	Unknown	88.8/72.7/70.6/87.2/113/119.2/100.7
439.1	2.4	Unknown	97.1/78.9/161.1/178.6
331	21.8–22.6	Unknown	169.1/125/331/168.1/124.9/313.1
293.1	269–27.1/27.7–27.8	Unknown	130.9/89/118.6/293.1/100.9/112.8/85.1
579.2	29-29.7/32.2–33.1/33.4–33.8/34.8-35.1	Unknown	289.1/245.2/579.2/ 426.8/409.4/453.2/288.8
441.1	42.2–42.5	Unknown	169/389.1/125/271.2/441.2
477	43.7–44	Unknown	301.2/476.8/113.1/175
463	43-43.1/43.3–43.6	Unknown	463.2/301.2/178.9/343.1/150.6 463.3/300.2/300.8/178.9/343
493.3	49.7/50.1–50.2/50.3-50.6	Unknown	315.2/447.4/161.1/131/493.3/447/160.7/314.9/447.4/315.1/130.6/161.2/118.9/179/191.2/447.3/149.2/179.1/131/88.9/118.9/161/191.2/315/251.3/221.1/47.4/149/493.3/179.1/101.2/160.9/89/118.8/130.6/190.7/251.1/221.1
507.2	50.3–50.9	Unknown	149.1/292.9/88.9/167.2/233.1/190.6/124.6/100.5/221.1/507.2

**Table 3:** Antifungal activity of pomace extract and PTB, free and loaded in poly(lactic-co-glycolic) acid nanoparticles on *Candida albicans* planktonic cells.

	MIC 50 (µg/mL)				
*C. albicans*	PTB	PTB + NPs	Pomace Extract	Pomace + NPs	NPs
10,231	>16	>16	12.5 ± 0.0	50 ± 0	>50
3135	>16	>16	6.25 ± 0.0	50 ± 0	>50
20,891	>16	>16	1.4 ± 0.4	12.5 ± 0.0	>50
10,261	>16	>16	12.5 ± 0.0	50 ± 0	>50
GM	>16	>16	6.06	35.35	>50
Median	>16	>16	9.45	50	>50
	**MIC 90 (µg/mL)**				
***C.albicans***	**PTB**	**PTB + NPs**	**Pomace Extract**	**Pomace + NPs**	**NPs**
10,231	>16	>16	25 ± 0	50 ± 0	>50
3135	>16	>16	25 ± 0	50 ± 0	>50
20,891	>16	>16	7.49 ± 3.05	50 ± 0	>50
10,261	>16	>16	21.87 ± 6.25	50 ± 0	>50
GM	>16	>16	17.76	50	>50
Median	>16	>16	25	50	>50
	**MIC 100 (µg/mL)**				
***C.albicans***	**PTB**	**PTB + NPs**	**Pomace extract**	**Pomace + NPs**	**NPs**
10,231	>16	>16	>50	>50	>50
3135	>16	>16	>50	>50	>50
20,891	>16	>16	>50	>50	>50
10,261	>16	>16	>50	>50	>50
GM	>16	>16	>50	>50	>50
Median	>16	>16	>50	>50	>50

MIC50, MIC90 and MIC100: The lowest concentration of extracts or PTB that caused growth inhibition ≥50%, ≥90% and 100%, respectively. SD = standard deviation. GM geometric mean. The values are expressed as mean MIC ± SD from three independent experiments in duplicate. PTB: pterostilbene. NPs: nanoparticles. NPs dimensions: 50 nm ± 10.

## Supplementary Materials

The following are available online at https://www.mdpi.com/1420-3049/24/11/2070/s1.
