# Supplementary material for: Anti-Candida Biofilm Activity of Pterostilbene or Crude Extract from Non-Fermented Grape Pomace Entrapped in Biopolymeric Nanoparticles
Source: Molecules. 2019 May 30;24(11):2070. doi: 10.3390/molecules24112070 (PMC6600237; doi:10.3390/molecules24112070)
Supplement: Supplementary file 1 [file molecules-24-02070-s001.pdf]

*Supporting Information*

# Anti-*Candida* Biofilm Activity of Pterostilbene or Crude Extract from Non-Fermented Grape Pomace Entrapped in Biopolymeric Nanoparticles

**Giovanna Simonetti [1,†], Cleofe Palocci [2,†], Alessio Valletta [3,†], Olga Kolesova [1], Laura Chronopoulou [2], Livia Donati [3], Antonio Di Nitto [2], Elisa Brasili[3], Pierpaolo Tomai [2], Alessandra Gentili [2] and Gabriella Pasqua [3,*]**

1 Department of Public Health and Infectious Diseases "Sapienza" University of Rome, P.le Aldo Moro 5, 00185 Rome, Italy; Giovanna.simonetti@uniroma1.it (G.S.); Olgakolesova@gmail.com (O.K.);
2 Department of Chemistry "Sapienza" University of Rome, 00185 Rome, Italy; cleofe.palocci@uniroma1.it (C.P.); laura.chronopoulou@uniroma1.it (L.C.); antonio.dinitto@uniroma1.it (A.D.N.); pierpaolo.tomai@uniroma1.it (P.T.); alessandra.gentili@uniroma1.it (A.G.)
3 Department of Environmental Biology "Sapienza" University of Rome, 00185 Rome, Italy; alessio.valletta@uniroma1.it (A.V.); donatilivia@gmail.com (L.D.); elisa.brasili@uniroma1.it (E.B.); gabriella.pasqua@uniroma1.it (G.P.)
* Correspondence: gabriella.pasqua@uniroma1.it; Tel: +39-06-49912414
† The authors equally contributed to the work

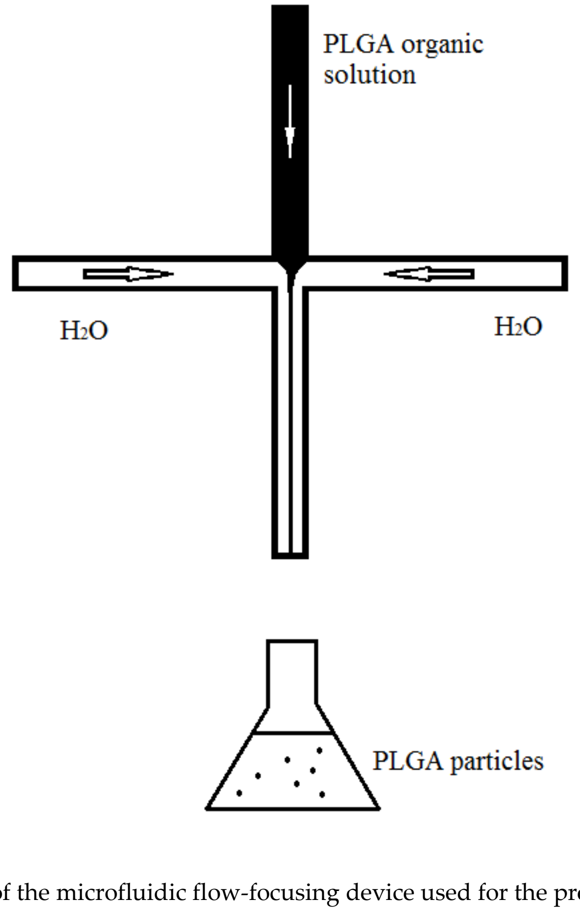


**Figure S1.** Schematic diagram of the microfluidic flow-focusing device used for the preparation of PLGA-based NPs.

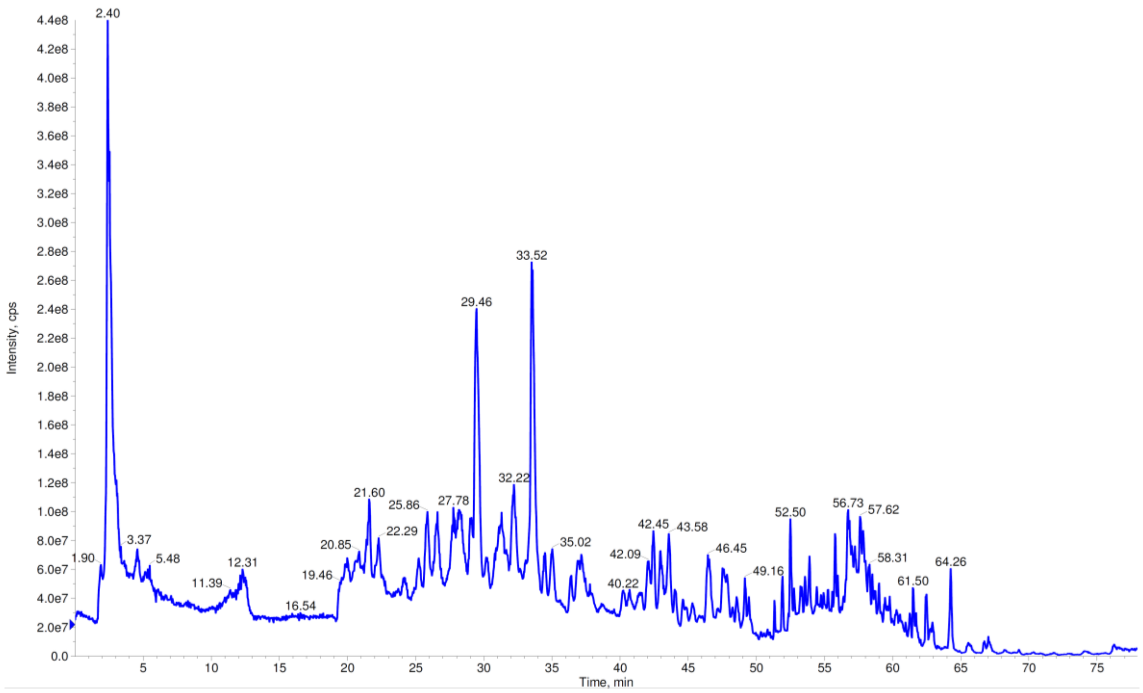


**Figure S2.** Total Ion Chromatogram (TIC) of the total extract from pomace before NP entrapment.

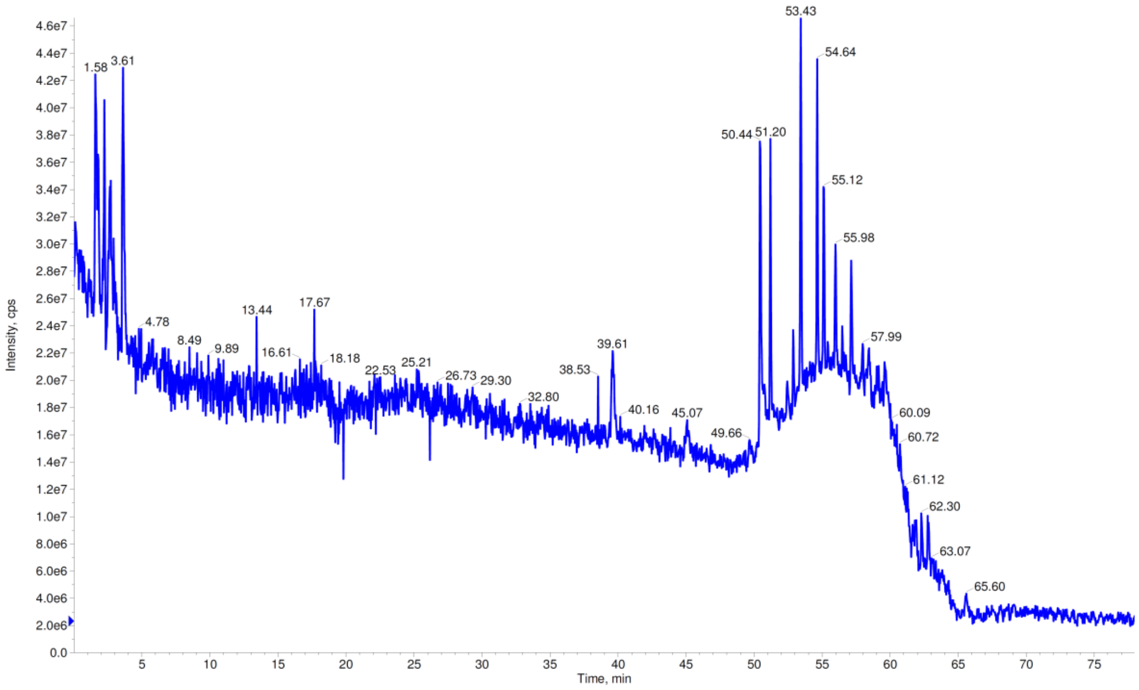


**Figure S3:** Total Ion Chromatogram (TIC) of the residual amounts of extract not entrapped.
